# H3K4me1 Distribution Predicts Transcription State and Poising at Promoters

**DOI:** 10.3389/fcell.2020.00289

**Published:** 2020-05-05

**Authors:** Sunhee Bae, Bluma J. Lesch

**Affiliations:** ^1^Department of Genetics, Yale School of Medicine, New Haven, CT, United States; ^2^Yale Cancer Center, Yale School of Medicine, New Haven, CT, United States

**Keywords:** histone, bivalent, poised, promoter, stem cell, germ cell, spermatogenesis, pluripotency

## Abstract

Monomethylation on lysine 4 of histone H3 (H3K4me1) is commonly associated with distal enhancers, but H3K4me1 is also present at promoter regions proximal to transcription start sites. To assess a possible role for H3K4me1 in dictating gene regulatory states at promoters, we examined H3K4me1 peak density around promoters in human and mouse germ cells using an analytic strategy that allowed us to assess relationships between different epigenetic marks on a promoter-by-promoter basis. We found that H3K4me1 exhibits either a bimodal pattern at active promoters, where it flanks H3K4me3, or a unimodal pattern at poised promoters, where it coincides with both H3K4me3 and H3K27me3. This pattern is correlated with gene expression level, but is more strongly linked to a poised chromatin state, defined by the simultaneous presence of H3K4me3 and H3K27me3, than to transcriptional activity. The pattern is especially prominent in germ cells, but is also present in other cell types, including embryonic stem cells and differentiated somatic cells. We propose that H3K4me1 is a key feature of the poised epigenetic state, and suggest possible roles for this mark in epigenetic memory.

## Introduction

Post-translational modifications on histone tails are closely correlated to transcriptional states. For example, trimethylation of histone H3 lysine 4 (H3K4me3) marks active gene promoters, while H3K9me3 marks regions subject to long-term repression (Barski et al., 2007). One such modification is monomethylation on lysine 4 of histone 3 (H3K4me1), a mark that has been linked to enhancers. Identifying regions enriched for H3K4me1 and depleted in H3K4me3, or regions enriched for both H3K4me1 and H3K27ac, have proven to be feasible methods for enhancer discovery (Heintzman et al., 2007; Creyghton et al., 2010). Mechanistic studies on the regulation of H3K4me1 marks have focused on the roles of MLL3 and MLL4, enzymes responsible for placing monomethylation on unmethylated lysine 4 which act primarily at enhancer regions (Guo et al., 2013; Hu et al., 2013; Dorighi et al., 2017).

At the same time, not all H3K4me1-enriched regions correspond to enhancers. H3K4me1 marks also exist at promoters, which implies that the H3K4me1 modification may have a context-dependent role in regulating transcription. Several studies have addressed questions about the role of H3K4me1 at promoters and highlighted the transcriptional features that may distinguish the functions of H3K4me1 at promoters compared to enhancers. H3K4me1 at promoters in the absence of H3K4me3 was associated with gene repression, while at active gene promoters H3K4me1 appeared to flank H3K4me3 in one study examining skeletal muscle cells (Cheng et al., 2014). Another study of mouse liver and pancreas likewise found that H3K4me1 signal flanks active promoters, although the relationship to H3K4me3 was not examined (Hoffman et al., 2010). An alternative view suggests that promoters and enhancers are a single class of transcriptional elements distinguished by different levels of transcription, and the varying H3K4me3 to H3K4me1 signal ratio at promoters compared to enhancers reflects the rates at which the elements recruit RNA Polymerase II (Core et al., 2014; Andersson et al., 2015).

Cell type is another aspect of regulatory context that may play a role in H3K4me1 function. Different modes of epigenetic regulation can take on larger or smaller roles in certain cell types. For example, embryonic stem cells (ESCs) and germ cells exhibit specialized regulatory mechanisms to maintain pluripotency and reprogramming potential. An epigenetic feature that is especially prominent in ESCs and germ cells is poised chromatin, which is defined by the simultaneous presence of two histone modifications, H3K4me3 and H3K27me3, at transcriptionally repressed promoters (Azuara et al., 2006; Bernstein et al., 2006; Mikkelsen et al., 2007). H3K4me3 at promoter regions is usually associated with active transcription, while H3K27me3 is associated with repression (Barski et al., 2007). The co-occurrence of these two opposing modifications at the same locus is thought to serve regulatory roles in ES cells and germ cells by preventing DNA methylation and preparing for resolution to active or more fully repressed states as cells differentiate (Azuara et al., 2006; Bernstein et al., 2006; Mikkelsen et al., 2007; Lesch and Page, 2014).

Here, we report distinct patterns of H3K4me1 that predict transcriptional regulatory states at promoters in germ cells and ESCs. We used an alternative approach to ChIP-seq data analysis in which we summarized chromatin signal around promoters based on ChIP-seq peak density instead of signal density. This approach allowed us to quantitatively interrogate histone modification patterns at a promoter-by-promoter level, in contrast to more commonly-used approaches that require pooling of multiple promoters to obtain average ChIP signals for a given mark. We used our approach to ask if patterns of H3K4me1 deposition near promoters could convey information about the regulatory state of the promoter. We examined ChIP-seq data for H3K4me1, H3K4me3, H3K27me3, and H3K27ac in mouse and human male germ cells, and found that H3K4me1 peak density around the transcription start sites (TSS) exhibits either a broad bimodal profile or a narrower unimodal profile centered at the TSS. We then examined the position of the H3K4me1 marks relative to H3K4me3, and found that unimodal H3K4me1 directly at the TSS predicts a poised state of chromatin, while bimodal H3K4me1 flanking the TSS predicts an active state. The bimodal distribution is not explained by nucleosome clearing, and the unimodal distribution is not explained only by low expression at these promoters. We conclude that unimodal H3K4me1 centered on the TSS is a characteristic feature of the poised epigenetic state in ESCs and germ cells.

## Materials and Methods

### Human Subjects

These studies were carried out in accordance with the Declaration of Helsinki. The protocol was approved by Yale University’s Human Subjects Institutional Review Board. Written informed consent was obtained from all subjects.

### Mouse Experiments

This study was carried out in accordance with the principles of the Basel Declaration and recommendations of Yale University’s Institutional Animal Care and Use Committee (IACUC). All procedures involving mice were approved by the Yale IACUC.

### Sample Collection and Sorting

Human testis samples were obtained from adult male patients undergoing vasectomy reversals at the Infertility Clinic of St. Louis. All men whose tissue was used in this study had a prior history of fertility demonstrated by at least one living child. Epididymal sperm quality and abundance proximal to the vasectomy site was assessed at the time of biopsy, and abundant, motile, morphologically normal sperm were confirmed for each patient. Testis biopsy samples were minced, dissociated using collagenase and trypsin, and then filtered to obtain a single-cell suspension as described (Bellvé, 1993). Mouse testes were isolated from adult CD1 males (Charles River Laboratories), and tissue from several mice was pooled before cell separation. Pachytene spermatocyte and round spermatid fractions were collected by StaPut (Shepherd et al., 1981; Bellvé, 1993; Liu et al., 2015), and pooled fractions were counted on a hemocytometer. Purity was >95% for each human sample and >90% for each mouse sample, as assessed by counts of 100 cells from each fraction under phase optics. Cells were washed once in PBS, fixed in 1% formaldehyde for 8 min at room temperature, and then quenched with 2.5 M glycine for 5 min at room temperature. Fixed cells were snap frozen in liquid nitrogen, then stored at −80°C.

### Chromatin Immunoprecipitation and Library Preparation

H3K4me3 and H3K27me3 data was previously published and is available on GEO (GSE68507). For H3K4me1 and H3K27ac ChIP, between 5 × 10^4^ and 5 × 10^6^ cells were used as starting material, depending on the number obtained from sample isolation and sorting. Pachytene spermatocytes and round spermatids were treated identically. For human spermatogenic cells, fixed cells frozen in lysis buffer (1% SDS, 10 mM EDTA, 50 mM Tris–HCl [pH 8]) were thawed on ice. For mouse spermatogenic cells, fixed cells frozen in PBS were thawed on ice, then washed once in cold PBS and resuspended in 100 ul lysis buffer. Once in lysis buffer, cells were incubated on ice for 5 min. 200 ul ChIP dilution buffer (0.01% SDS, 1.1% Triton X-100, 1.2 mM EDTA, 16.7 mM Tris–HCl [pH 8], 167 mM NaCl) was then added to each sample. Samples were sonicated in aliquots of 150 ul in 0.5 ml Eppendorf tubes at 4C using a BioRuptor (Diagenode) for 35 cycles on High setting, 30 s on/30 s off. Aliquots of the same sample were then re-pooled and spun down at 12,000 × *g* for 5 min, and the supernatant moved to a fresh tube. Chromatin from each sample was then split into two separate tubes (150 ul in each), and 700 ul dilution buffer, 50 ul lysis buffer, and 100 proteinase inhibitor cocktail (Complete Mini tablets, Roche #11836153001) were added to each tube. 50 ul of each sample was set aside as input. The remainder of the ChIP was performed as previously described (Lesch et al., 2013), except that the second wash for H3K27ac samples was performed in high-salt immune complex wash buffer (0.1% SDS, 1% Triton X-100, 2 mM EDTA, 20 mM Tris–HCl (pH 8.1), 500 mM NaCl) instead of low-salt immune complex wash buffer. Immunoprecipitation was performed using 1.0 ug of antibody to H3K4me1 (Abcam #ab8895, RRID:AB_306847) or 1.0 ug of antibody to H3K27ac (Abcam #ab4729, RRID:AB_2118291).

### Sequencing Library Preparation and Sequencing

ChIP libraries were prepared using a TruSeq ChIP sample prep kit (Illumina), according to the manufacturer’s instructions, except that size selection was performed after (instead of before) PCR amplification. All libraries were sequenced on an Illumina HiSeq2500 with 40-base-pair single-end reads.

### ChIP-seq Data Processing

Image analysis and base calling were done with the standard Illumina pipeline for HiSeq2500. Data was quality-filtered using fastq_quality_filter from the FASTX toolkit (RRID:SCR_005534) with the following parameters: -q 20 -p 80. ChIP-seq data was aligned to either the mouse (mm10) or human (hg19) genome using Bowtie2 in –end-to-end –fast mode with default settings (Langmead and Salzberg, 2012, RRID:SCR_005476). Peaks were called using MACS2 with the following parameters: narrowPeak, *q* = 0.1 (H3K4me3); broadPeak, *q* = 0.2 (H3K4me1 from mouse PS and RS); broadPeak, *q* = 0.1 (all other data) (Zhang et al., 2008, RRID:SCR_013291). *q*-values for peak calling were selected based on reconciliation of peak boundaries with ChIP signal visualized on the UCSC genome browser.

### RNA-seq Data Processing

RNA-seq data was processed using kallisto (Bray et al., 2016, RRID:SCR_016582) with the following parameters: –bias -b 40 -t 8. Ensembl transcripts (cDNA and ncDNA) from mm10 or hg19 were used to create reference indexes for each species (Cunningham et al., 2019).

### Data Analysis

Density plots were generated using custom R scripts. First, the center of each peak ascertained by MACS2 was obtained and the distance from the center of the peak to the nearest TSS was calculated. The distance values were used as input to calculate a density distribution using a Gaussian smoothing function with bandwidth of 15, and the resulting probability function was plotted in R. Heatmaps were generated using the ggplot2 package in R (Wickham, 2011, RRID:SCR_014601) and custom R scripts. For each TSS, its distance to the nearest H3K4me1 and H3K4me3 was calculated, and the two distance values per TSS were used to generate a heatmap based on 2d bin counts using geom_bin2d() in ggplot2.

To obtain a list of poised promoter regions, a list of H3K4me3 peaks that overlap H3K27me3 peaks were generated with BEDTools using -wa option (Quinlan and Hall, 2010, RRID:SCR_006646). To obtain H3K4me1 peaks at poised promoters, a list of H3K4me1 peaks with overlaps with poised H3K4me3 was generated again with -wa option on BEDTools.

Violin plots were generated using ggplot2 and custom R scripts. TPM values obtained through kallisto were used to categorize the TSS based on expression level, using the following categories: TPM ≤ 1, 1 < TPM ≤ 5, 5 < TPM ≤ 10, TPM > 10. For each TSS, a category based on TPM value was assigned, and its distance to the center of the nearest H3K4me1 peak was calculated. The distances in each TPM category were used to create violin plots using geom_violin() and geom_boxplot() in ggplot2.

Statistical comparisons of multi-group, non-parametric data were performed using the Kruskal-Wallis rank sum test with the kruskal.test() function in R.

### Data Availability

All germ cell datasets are available on GEO under accession numbers GSE68507 (H3K4me3 ChIP-seq, H3K27me3 ChIP-seq, and RNA-seq) and GSE145225 (H3K4me1 and H3K27ac ChIP-seq). Other public datasets used in this study are from mouse myeloid cells (GSE85072), fetal liver (GSE119201), and adult kidney (GSE31039).

### Code Availability

Custom R and Python scripts used for this study are available on Github^1^.

## Results

### Distribution of H3K4me1 Peaks Near Promoters Differs From Other Histone Marks

**FIGURE 1:**
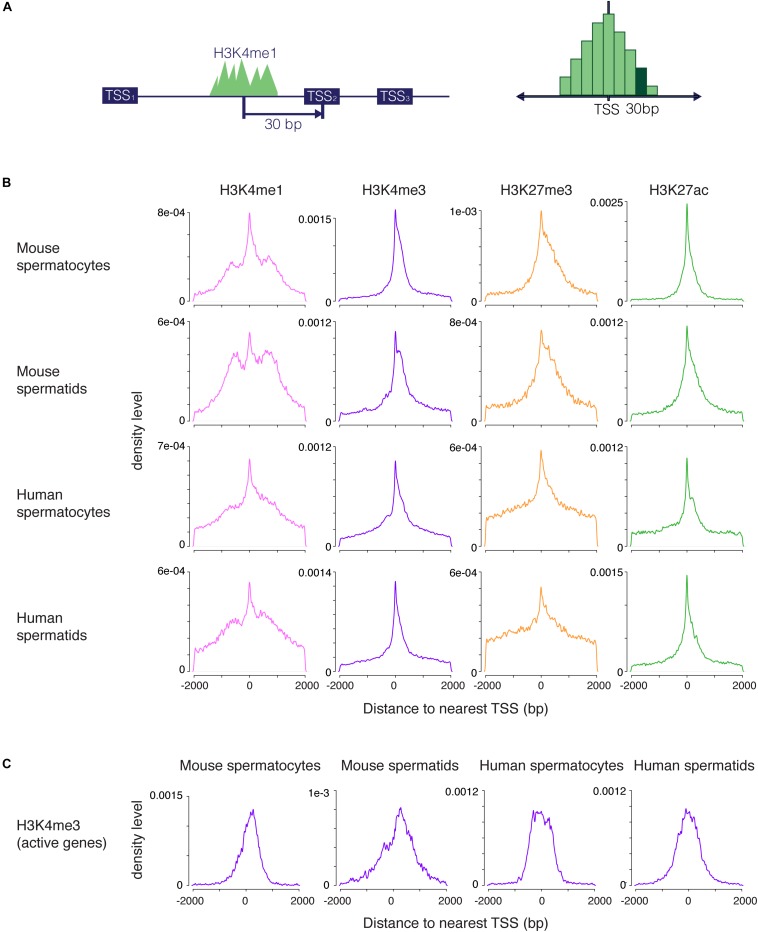
Distribution of H3K4me1, H3K4me3, H3K27me3, and H3K27ac peaks near promoters. **(A)** Scheme for generating density plots. For each peak, the distance from the peak center to its nearest transcription start site (TSS) was obtained, and a density distribution was calculated from the distance values. **(B)** Density plots for four histone modifications in mouse and human male germ cells at two stages of spermatogenesis. All four marks have a unimodal set of peaks centered at the TSS, but only H3K4me1 has an additional bimodal peak density displaced from the TSS. **(C)** Density distribution of H3K4me3 peaks at highly transcribed (tpm > 10) TSS. The density plots do not exhibit profiles that indicate nucleosome clearing. bp, base pairs.

We set out to examine H3K4me1 distribution in germ cells and embryonic stem cells (ESCs), cell types that share many regulatory mechanisms related to reprogramming and pluripotency. To evaluate the distribution of H3K4me1 at promoters relative to other histone modifications, we called peaks from ChIP-seq data for H3K4me1 and H3K27ac (this study), and from H3K4me3 and H3K27me3 (Lesch et al., 2016) from mouse and human spermatogenic cells at two developmental stages, pachytene spermatocytes and round spermatids. Pachytene spermatocytes are undergoing the first meiotic division, while round spermatids are haploid germ cells that have completed meiosis but have not yet differentiated into sperm. These two cell types represent two very different cellular states, but share gene regulatory features characteristic of germ and stem cells (Lesch et al., 2013; Hammoud et al., 2014). We calculated the base pair unit distance from each peak to the nearest TSS and visualized the distribution of peak distances with a density plot, where the *y* axis shows the probability that the nearest peak is centered a given distance away from the TSS (Figure 1A). H3K4me3, H3K27me3 and H3K27ac peak distances were all centered within 1kb of the TSS, and the distributions of the three marks were unimodal, as expected for these promoter-centric modifications. In contrast, H3K4me1 exhibited a mixed unimodal and bimodal pattern in which a large fraction of peaks were centered 300–1000 bp away from the nearest promoter region. The bimodal pattern was unique to H3K4me1 and was present across all cell types examined, although it appeared less pronounced in human compared to mouse cells (Figure 1B).

We considered several possible explanations for the unique distribution of H3K4me1 peaks around promoters. First, most H3K4me1 signal might be coming from enhancers, as has been previously described. If so, peak distribution would be centered away from the TSS, but we would expect to see a broad distribution of H3K4me1 peaks extending away from the TSS in both directions, rather than the accumulation in density we observe in the 300–1000 bp range. Second, the bimodal profile could result from nucleosome clearing at active promoters, similar to the pattern commonly seen in metagene plots of H3K4me3 signal (Barski et al., 2007). This explanation has been previously proposed in the context of bimodal H3K4me1 profiles (Hoffman et al., 2010). However, our method does not detect nucleosome clearing for H3K4me3 at active TSS, where this phenomenon is known to occur (Figure 1C), indicating that our method of analysis does not reveal nucleosome-free regions. Finally, the unimodal distributions we observed for other marks indicate that a bimodal pattern is not simply an artifact of our analysis method. We conclude that H3K4me1 occupies a distinctive distribution at promoters in addition to its well-studied role at distal enhancers (Rickels et al., 2017; Local et al., 2018).

### The Bimodal Distribution Is Characteristic of H3K4me1 Across Cell Types

**FIGURE 2:**
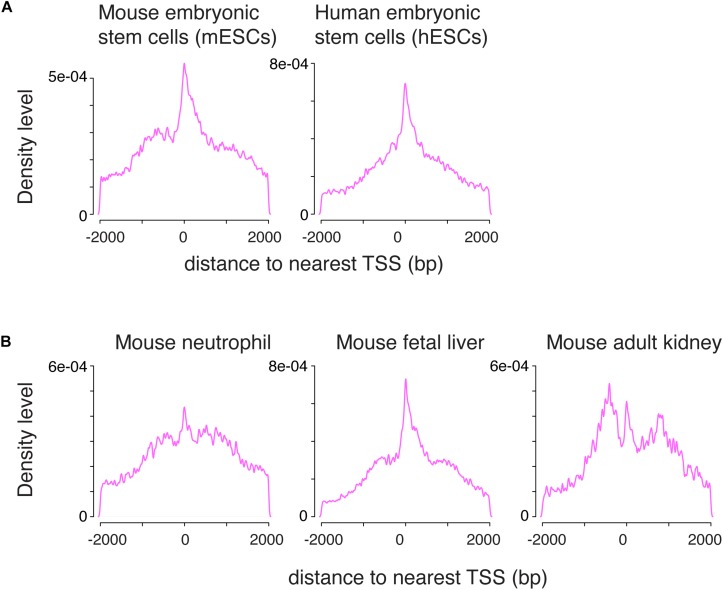
The combination of unimodal and bimodal H3K4me1 distributions is observed across various cell types. **(A)** Density distribution in mouse and human embryonic stem cells. A mixed unimodal and bimodal pattern of H3K4me1 is present, similar to germ cells. **(B)** Density distribution in mouse neutrophils, fetal liver, and adult kidney. The mix of unimodal and bimodal distributions is observed in all three cell types, but relative prominence of the unimodal H3K4me1 distribution varies. bp, base pairs.

To determine whether the pattern of H3K4me1 distribution around the TSS was unique to germ cells, we applied the same data-processing protocol to published ChIP-seq data from mouse embryonic stem cells (mESCs) and human embryonic stem cells (hESCs) (Rada-Iglesias et al., 2011; Garland et al., 2019). The mixed unimodal/bimodal pattern of H3K4me1 density around the TSS was recapitulated in mESCs, and to a lesser degree in hESCs (Figure 2A). We then examined somatic cells across several lineages using public datasets, and found that similar mixed unimodal/bimodal H3K4me1 patterns are present in mouse myeloid cells, fetal liver, and adult kidney cells (Figure 2B; Encode Project Consortium, 2012; Kotzin et al., 2016). Whereas both the unimodal and bimodal H3K4me1 patterns in fetal liver closely recapitulated the pattern observed in germ cells, the unimodal TSS probability density was dampened relative to the bimodal density in myeloid and kidney cells. We postulate that the bimodal H3K4me1 pattern is shared among all cell types, while the unimodal pattern is more accentuated in cell types that have pluripotent or multipotent potential.

### H3K4me1 Distribution Pattern Correlates to Expression Level

**FIGURE 3:**
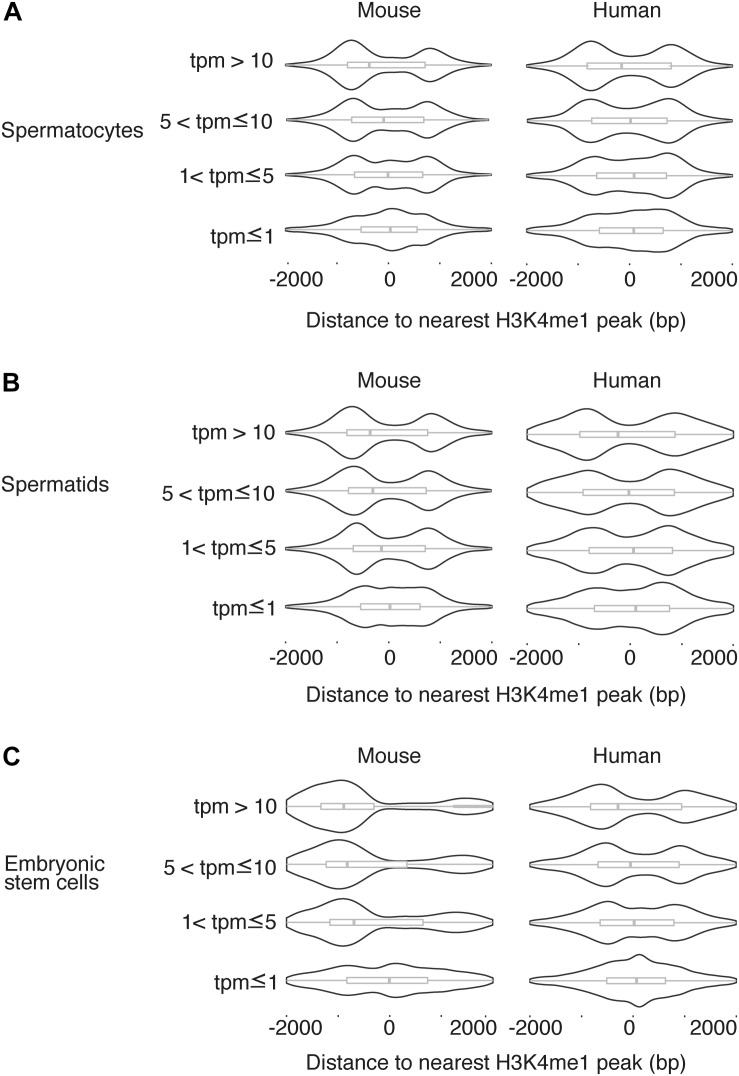
H3K4me1 distribution pattern is correlated to expression level. Distribution of H3K4me1 with respect to the TSS in mouse and human pachytene spermatocytes **(A)**, round spermatids **(B)**, and embryonic stem cells **(C)**, classified by transcript level. Box plots enclosed within the violin plots show the median (vertical line), interquartile range (box) and total range within the plot boundaries (horizontal line) for the distance between the H3K4me1 peak and TSS. In all cell types, TSS with lower expression are associated with H3K4me1 peak densities directly overlapping the TSS, while TSS with higher expression are associated with H3K4me1 peak densities that flank the TSS. Comparisons across the four categories of transcript levels were significant at a threshold of *p* < 0.05 by the Kruskal-Wallis test for all mouse cell types (spermatocyte *p* = 2.395e-5, spermatid *p* = 4.368e-6, ESC *p* < 2.2 e-16) and for human spermatocytes (*p* = 1.915e-6). Comparisons in human spermatids and human ESCs were not significant (*p* > 0.05). bp, base pairs. tpm, transcripts per million.

We next asked whether the H3K4me1 distribution around the TSS was related to gene expression level. For each TSS in the mouse genome, we found the distance to the nearest H3K4me1 peak, then grouped the TSS into four categories based on expression level as measured by RNA-seq (Lesch et al., 2016) (see section “Materials and Methods”). We observed a consistent trend in which H3K4me1 peaks overlap the TSS with a unimodal distribution at genes with lower expression levels, and flank the TSS with a bimodal distribution at genes with higher expression (Figure 3). The difference in distribution among expression levels was statistically significant for all cell types tested in mouse and for human pachytene spermatocytes (*p* < 0.05, Kruskal-Wallis test). The same trend is present in human round spermatids and hESCs, although the distribution differences were not statistically significant. Lack of statistical significance in the latter two cell types may be due to increased noise in the datasets, although we cannot exclude a true biological difference in histone mark distributions.

### H3K4me1 Distribution Defines Two Categories of H3K4me3-Positive Promoters

Because H3K4me1 is an intermediate molecular state between unmethylated H3K4 and H3K4me3, we considered the possibility that H3K4me1 distribution at promoters is purely a byproduct of mechanisms that directly regulate H3K4me3. In this scenario, H3K4me1 would be expected to mark genes in transition between expression states: either gain of H3K4me3 along with gain of transcription, or loss of H3K4me3 along with loss of transcription.

**FIGURE 4:**
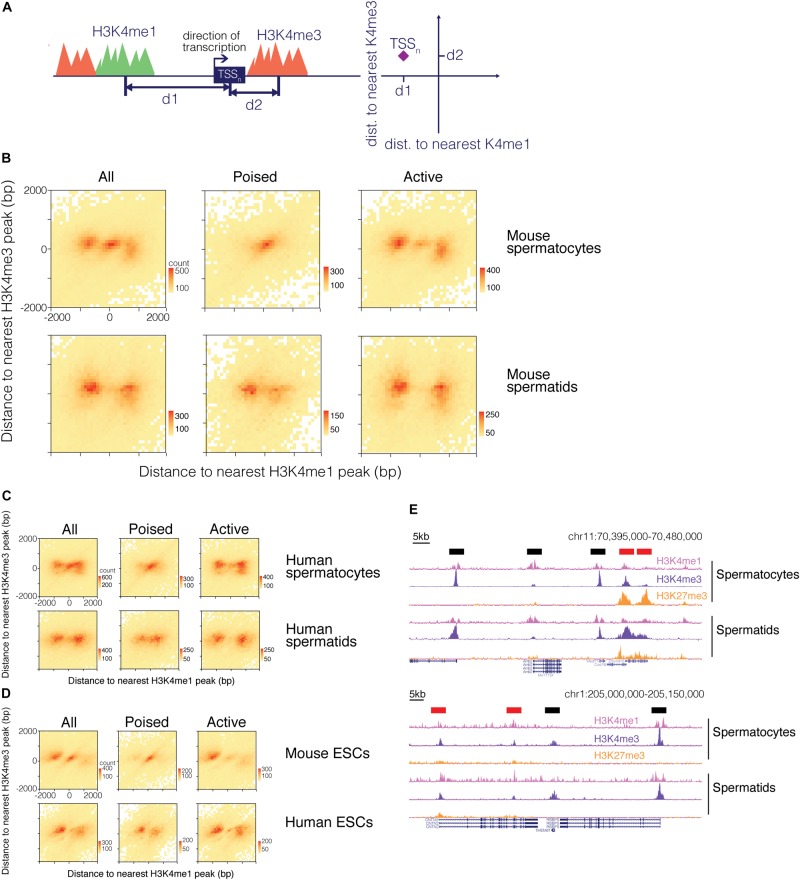
H3K4me1 distribution around H3K4me3 predicts the epigenetic state of promoters. **(A)** Scheme for mapping TSS with respect to both H3K4me1 and H3K4me3 marks. For each TSS, distances to its nearest H3K4me1 peak and its nearest H3K4me3 peak were calculated, and the TSS were classified based on distances to the two histone modifications. **(B)** Heatmap of TSS distribution in mouse pachytene spermatocytes and round spermatids. Most TSS were concentrated at either the center of the heatmap or both sides of the center group to form “wings”. The center cluster and the wings separate when the TSS are grouped based on poising. **(C)** Heatmap of TSS distribution in human germ cells. **(D)** Heatmap of TSS distribution in mouse and human embryonic stem cells. **(E)** ChIP-seq signal tracks at poised and active promoters in mouse (top) and human (bottom) germ cells. H3K4me1 signal at active promoters (black bars above signal tracks) flanks H3K4me3 signal while H3K4me1 signal at poised promoters (red bars above signal tracks) exhibits a unimodal profile. bp, base pairs.

We therefore examined how the H3K4me1 pattern relates to the H3K4me3 pattern around promoter regions. We calculated the distance to both the nearest H3K4me1 peak and the nearest H3K4me3 peak for each TSS (Figure 4A). We visualized the distribution of the TSS with respect to surrounding H3K4me1 and H3K4me3 marks as a heatmap, where the total number of data points represented in the heatmap is equal to the number of TSS that neighbor a H3K4me1 and a H3K4me3 region within 2000bp. A clear pattern emerged from this analysis, in which the TSS either grouped at the center of the heatmap or into “wings” on both sides of the center cluster (Figure 4B). The TSS occupying the center of the plot represent promoters with both H3K4me1 and H3K4me3 centered on the TSS, while the group of TSS that form the “wings” correspond to promoters that have only H3K4me3 directly at the TSS and are flanked by H3K4me1. This finding indicates that TSS marked by H3K4me3 can be classified into two groups based on the distribution of nearby H3K4me1 signal, and implies that H3K4me1 distribution is not purely dictated by the distribution of H3K4me3 at a given promoter.

### H3K4me1 Pattern Separates Poised From Active Promoters

Based on our finding that transcription is related to the shape of the H3K4me1 distribution, we hypothesized that the two patterns observed in our joint analysis might represent poised and active promoters. Active promoters are H3K4me3 positive, H3K27me3 negative, and transcriptionally active, while poised promoters are marked by both H3K4me3 and H3K27me3 and are transcriptionally repressed. We sorted the TSS into “poised” and “active” groups based on whether or not the H3K4me3 region nearest a given TSS overlapped with H3K27me3 signals. When poised and active TSS were plotted separately, we found that the pattern observed in our first heatmap resolved into separate bimodal and unimodal patterns based on whether the TSS is active or poised, respectively (Figures 4B–D). These two patterns are identifiable in the ChIP-seq signal tracks: H3K4me1 is centered at the TSS at poised, H3K4me3/H3K27me3-positive promoters, while at active gene promoters H3K4me1 “clears” from TSS bearing high H3K4me3 signals to flank the H3K4me3 marks (Figure 4E). The H3K4me1 signal pattern at active promoters helps to explain the asymmetry we observe in the bimodal distribution of H3K4me1 in mouse ESCs, in which a higher proportion of H3K4me1 regions occur upstream of the TSS (Figure 3C). H3K4me3 peaks are often centered downstream of the TSS at active genes, and when bimodal H3K4me1 regions symmetrically flank a H3K4me3 region at a TSS, the upstream H3K4me1 peak will therefore be positioned closer to the TSS than the downstream peak. Since our method of analysis records the single closest H3K4me1 peak for each TSS, the H3K4me1 peak upstream of the TSS is more likely to be recorded. This asymmetry is particularly pronounced in mouse ESC, possibly due to higher resolution of the sequencing data, but is also present in the H3K4me1 distribution for other cell types.

In all three cell types from mouse, heatmaps of TSS reflected a distribution in which H3K4me1 directly occupied promoter regions of poised genes and separated to border the H3K4me3 signal in active promoters. A similar phenomenon was observed for human germ cells and ESCs. While H3K4me1 marks did not always directly overlap the TSS of poised genes in human round spermatids and ESCs, the marks localized closer to the TSS for poised genes compared to active genes (Figures 4C,D).

### Poising Is Dominant Over Expression Level in Determining H3K4me1 Peak Profile

**FIGURE 5:**
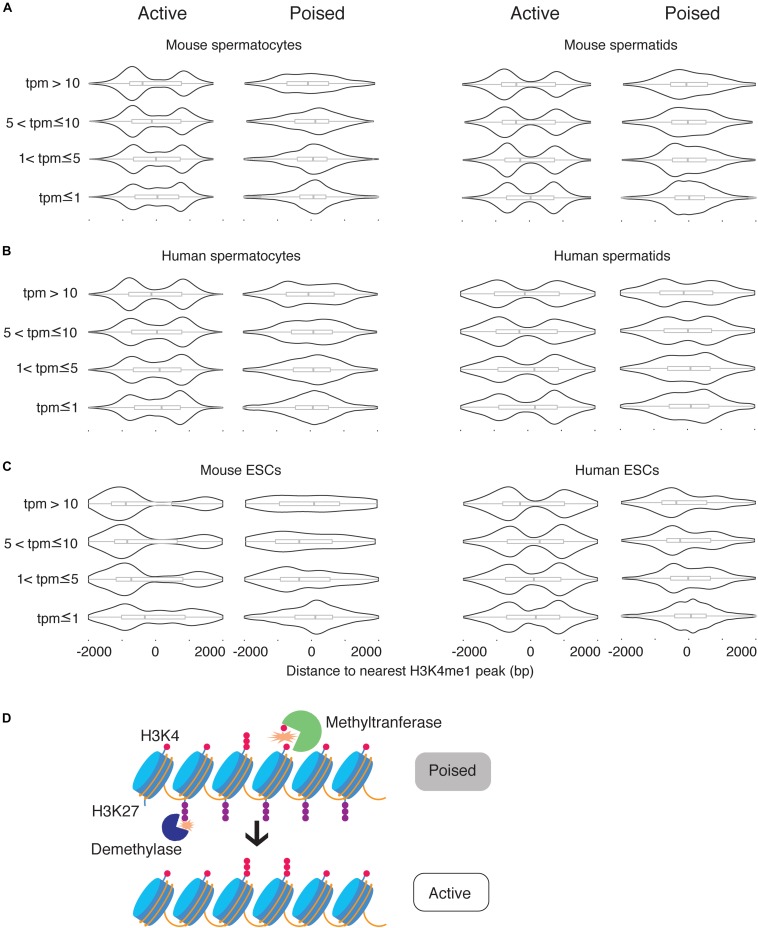
H3K4me1 peak profile correlates best with epigenetic poising at promoters. **(A)** Distribution of H3K4me1 with respect to the TSS at active promoters and poised promoters in mouse spermatocytes and spermatids, classified by expression level. **(B)** Distribution of H3K4me1 in human spermatocytes and spermatids, classified by expression levels. **(C)** Distribution of H3K4me1 in mouse and human ESCs. Box plots enclosed within the violin plots show the median (vertical line), interquartile range (box) and total range within the plot boundaries (horizontal line) for the distance between the H3K4me1 peak and TSS. All comparisons between active promoters and poised promoters were significant (*p* < 0.05) by the Kruskal-Wallis test. **(D)** Model for the role of H3K4 monomethylation in activation of poised promoters. Preexisting H3K4me1 is further methylated to H3K4me3, while H3K27me3 is removed from the promoter region.

The correlations between H3K4me1 distribution, poised chromatin state, and gene expression level raised two possibilities for the role of H3K4me1: H3K4me1 could be functionally related to expression level and correlated with poised chromatin, or H3K4me1 could be functionally related to the poised histone modification state and correlated with gene repression. To discriminate between the two possibilities, we first obtained a list of H3K4me3 peaks that overlap H3K27me3 peaks (“poised” peaks). Then we calculated the distances between the TSS and the nearest H3K4me1 peak and grouped the TSS based on whether the nearest H3K4me1 peaks overlapped with poised peaks. TSS where the nearest H3K4me1 peak overlapped with a poised peak were classified as poised TSS. We then defined epigenetically ‘active’ TSS as TSS where the nearest H3K4me1 peak did not overlap a poised peak, and the nearest H3K4me3 peak was within 500bp of the TSS (see section “Materials and Methods”). Then, each of the two categories of TSS were further classified into four groups based on expression levels, using the same thresholds for transcription level that we used in our previous analysis. As expected, poised promoters were more likely to be silent (tpm = 0) compared to epigenetically active genes, and when expressed their transcript levels were significantly lower than expressed epigenetically active genes (Supplementary Figure S1). However, there were enough poised genes with high expression levels and epigenetically active genes with low expression levels to test for statistical significance (*n* ≥ 167 for all categories and datasets). We found that the unimodal, promoter-centered H3K4me1 distribution marked poised promoters regardless of expression level, while a bimodal H3K4me1 distribution marked epigenetically ‘active’ (H3K4me3-only) promoters regardless of expression level (Figures 5A–C). We conclude that the promoter-centered, unimodal distribution of H3K4me1 is a feature of the poised state.

## Discussion

We identified two distinct H3K4me1 distribution patterns at promoters in mouse and human germ cells and ESCs by examining the density of H3K4me1 peaks around transcription start sites. We found a unimodal distribution of peaks at one set of promoters, corresponding to H3K4me1 occupying the promoter region directly at the TSS. A different set of promoters is associated with a bimodal peak density that corresponds to H3K4me1-enriched regions flanking the TSS. These distributions corresponded to alternative regulatory states. Unimodal H3K4me1 signal was correlated with lower transcript expression and the presence of poised (H3K4me3/H3K27me3 bivalent) chromatin, while the bimodal H3K4me1 pattern was linked to high expression level and the absence of poising. By examining promoters where chromatin state and expression level were discordant, we found that H3K4me1 patterns were more strongly associated with the chromatin state dictated by H3K4me3 and H3K27me3 than with the expression level of the promoter.

The observation that H3K4me1 signal exhibits two different patterns around promoters has been previously reported in mouse muscle cells *in vitro* and in adult mouse pancreatic islet and liver cells, as well as a handful of other cell types (Hoffman et al., 2010; Cheng et al., 2014). We found that these two promoter-associated H3K4me1 patterns are also present in germ cells. In contrast to these previous studies, we found that the bimodal pattern of H3K4me1 is independent of nucleosome clearing, and instead may signify a more direct regulation of the balance between H3K4me1 and H3K4me3. In addition, we associate the unimodal H3K4me1 distribution directly with the bivalent H3K4me3/H3K27me3 histone modification state. In muscle cells, H3K4me1 was found to occupy repressed H3K27me3-positive promoters, and similar findings have been reported for brown adipocytes and hESCs (Cheng et al., 2014; Dozmorov, 2015; Brunmeir et al., 2016). While these studies found that H3K4me1 correlates with H3K27me3 alone and acts as a repressive mark, we find that unimodal H3K4me1 patterns correlate strongly with poised promoters marked by both H3K4me3 and H3K27me3. This finding suggests that H3K4me1 is an essential feature of poised chromatin in cells with pluripotent and reprogramming potential, such as ESCs and germ cells. Alternatively or in addition, H3K4me1 may act as either a “memory” or precursor of poising at transiently-poised promoters in these populations.

The latter possibility has been explored through the study of “Placeholder” nucleosomes, which are characterized by H3K4me1 and H2A. Z in zebrafish sperm. These specialized nucleosomes deter DNA methylation at genomic regions they occupy until the regions acquire poising or activating marks at zygotic genome activation (Murphy et al., 2018). H3K4me1 and H2A. Z serve as a transient chromatin state that passively drives demethylation of DNA without activating transcription while DNA methylation patterns are reprogrammed. We speculate that the H3K4me1 we observe at poised promoters in human and mouse germ cells serves a similar regulatory function: it maintains an epigenetically neutral state at promoters that are important for development and differentiation. Interestingly, H2A. Z is also enriched at poised promoters in mESCs and hESCs (Ku et al., 2012).

Why do pluripotent systems incorporate H3K4me1 at poised promoters? Structure studies have shown that DNMT3L directs the DNA methyltransferases DNMT3a and DNMT3b to nucleosomes bearing unmethylated H3K4, and that monomethylation is sufficient to deter binding of histone-interacting domain of DNMT3L (Ooi et al., 2007). H3K4me1 could serve to broadly demarcate regions of the DNA that must be kept hypomethylated during spermatogenesis and early embryogenesis. H3K4me1 can prevent DNA methylation without recruiting transcription-activating regulators, keeping these regions in a transcriptionally neutral state. In this respect, H3K4me1 is an ideal molecular marker to carry “memories” of transcription. This view of H3K4me1 may also help explain the bimodal H3K4me1 distribution we observed at promoters of active genes. H3K4me1 would appear to flank H3K4me3 peaks at promoter regions if the H3K4me1 marks are replaced with H3K4me3 proximal to the TSS as transcription is activated (Figure 5D).

Interpretation of H3K4me1 as transcriptional memory also has interesting implications for the mechanisms underlying epigenetic inheritance. When DNA is replicated, parent histones are thought to be distributed to one of the daughter strands, which results in a “dilution” of histone modifications (Alabert et al., 2015). At the same time, H3K4me3 regions must be kept narrowly focused at the TSS: spreading of H3K4me3 is associated with defects in transcriptional activation (Kidder et al., 2014). Narrowly H3K4-trimethylated regions may therefore be necessary for accurate control of transcription, but also endanger inheritance of transcriptional memory. Broad H3K4me1 regions could overcome this dilemma: a broad stretch of H3K4me1 is more likely to be “remembered” by both strands of the replicated DNA and would also maintain a chromatin state that is permissive for recruitment of the transcriptional machinery. While additional experiments need to be done to validate the role of H3K4me1 in regulating H3K4me3 deposition, our findings provide a new perspective in interpreting the role and regulation of H3K4me1 marks at promoters.

## Data Availability Statement

The datasets generated for this study can be found in the NCBI Gene Expression Omnibus (GEO) under accession number GSE145225.

## Ethics Statement

The studies involving human participants were reviewed and approved by the Yale University Human Subjects Institutional Review Board. The patients/participants provided their written informed consent to participate in this study. The animal study was reviewed and approved by the Yale University Institutional Animal Care and Use Committee.

## Author Contributions

SB wrote the code, designed and performed the analysis, interpreted the data, and wrote the manuscript. BL performed the ChIP experiments, contributed to data interpretation, and contributed to writing the manuscript.

## Conflict of Interest

The authors declare that the research was conducted in the absence of any commercial or financial relationships that could be construed as a potential conflict of interest.

## Footnotes

1 https://github.com/Lesch-Lab/H3K4me1-profiles
