# Supplementary figures and images for: H3K4me1 Distribution Predicts Transcription State and Poising at Promoters
Source: Front Cell Dev Biol. 2020 May 5;8:289. doi: 10.3389/fcell.2020.00289 (PMC7214686; doi:10.3389/fcell.2020.00289)

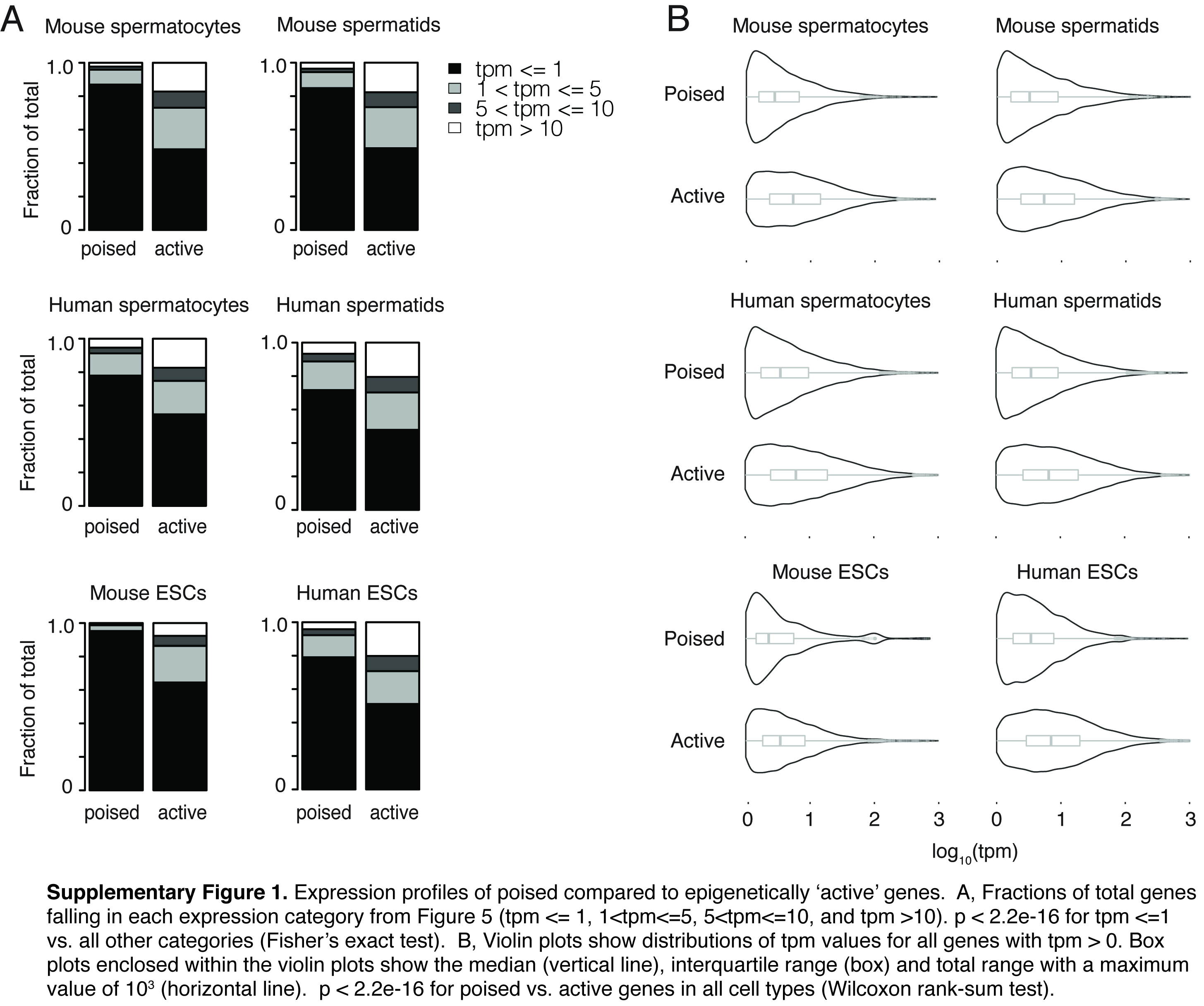

Supplement: Supplementary file 1 [file Image_1.tif]
